# Detection of *Toxocara cati* Larvae in a Common Buzzard (*Buteo buteo*) and in a Red Kite (*Milvus milvus*) in Basilicata Region, Italy

**DOI:** 10.3390/ani12060710

**Published:** 2022-03-11

**Authors:** Mariateresa Toce, Antonella Cristina Romano, Ileana Pietragalla, Gianluca Marucci, Lucia Palazzo

**Affiliations:** 1 Istituto Zooprofilattico Sperimentale della Puglia e della Basilicata, Via Manfredonia 20, 71121 Foggia, Italy; mariateresa.toce@izspb.it (M.T.); ileana.pietragalla@izspb.it (I.P.); lucia.palazzo@izspb.it (L.P.); 2 Unit of Foodborne and Neglected Parasitic Diseases, Department of Infectious Diseases, Istituto Superiore di Sanità, Viale Regina Elena 299, 00161 Rome, Italy; gianluca.marucci@iss.it

**Keywords:** *Toxocara cati*, *Buteo buteo*, common buzzard, red kite, *Milvus milvus*, paratenic host, zoonosis

## Abstract

**Simple Summary:**

In this study we report the detection of *Toxocara cati* larvae in the muscle tissue of two birds of prey, a red kite (*Milvus milvus*) and a common buzzard (*Buteo buteo*), received in our laboratory as part of the Wildlife Monitoring and Control Plan of the Basilicata Region (Italy). To the authors’ knowledge, this is the first report of identification of *T. cati* larvae in these two species.

**Abstract:**

*Toxocara cati* is a common parasite of wild and domestic felines, and presents a cosmopolitan distribution. Adult parasites localize in the gut of the definitive host giving rise to the infection, which usually runs asymptomatic. These worms produce eggs that are excreted with feces into the environment, where they become a source of infection for paratenic hosts, such as mammals, birds, and invertebrates. In this brief communication, we report the detection of *T. cati* larvae in a common buzzard (*Buteo buteo*) and a red kite (*Milvus milvus*), in the Basilicata Region of Italy. This result may be important to define new pathways of spread and survival of *T. cati* in the wild.

## 1. Introduction

*Toxocara cati* is an ascarid nematode in the order Ascarididia, superfamily Ascaridoidea, family Toxocaridae. Adult forms of the parasite live in the upper tract of the small intestine of their definitive hosts, felids. Female worms can produce up to 200,000 eggs per day. Eggs passed in feces are not infectious, and require an incubation period of 1–4 weeks, depending on temperature, in the soil to embryonate [1]. Eggs containing third stage larva can remain infectious in the environment for months or years [2]. After being ingested by the cat, the eggs hatch in the small intestine and release larvae that perforate the intestinal wall and migrate to the liver and lungs via the bloodstream. From the lungs, the larvae ascend into the trachea, and, by ingestion, reach the intestine. In the small intestine, the larvae develop to adults and release eggs that are excreted with feces. The transmission of *T. cati*, in addition to the fecal–oral route, can also occur via trans–mammary transmission when the female is infected during late pregnancy. Vertical transmission is, instead, absent for *T. cati*, contrary to *T. canis*, in which it represents the major source of contamination [3]. In paratenic hosts, development into the adult stage does not occur, and infectious larvae persist in tissues in a developmentally arrested stage [4]. Paratenic hosts harboring infective larvae in their tissues play an important role in *T. cati* diffusion, since, when ingested by a definitive host, the larvae may complete their final molt to evolve into adult worms. In Italy, the distribution of *T. cati* is ubiquitous in cat populations. Multicenter studies, conducted in Italy, on the overall prevalence of gastrointestinal nematodes, have found that *T. cati* is the most prevalent species in naturally infected cats from feral colonies, shelters, and private households, and that the overall prevalence of *T. cati* infection is significantly more frequent in cats aged < 1 year [5]. Humans can become infected through accidental ingestion of embryonated eggs found in contaminated soil or food, or by ingestion of undercooked meat containing the larvae. *T. cati* larvae, after ingestion, can migrate into a variety of tissues and cause clinical manifestations such as visceral larva migrans (VLMs), ocular larva migrans (OLMs), covert or common toxocariasis (CT), and neurotoxocariasis (NT) [6]. Most clinical manifestations induced by *T. cati* infection run asymptomatically or nonspecifically; therefore, its impact on public health may be underestimated [7,8].The red kite is a diurnal raptor belonging to the Accipitridae family; population estimates offer a total picture of approximately 25,200–33,400 pairs, concentrated essentially throughout Europe. In Italy, at present, the species is discontinuously distributed in the central–southern and insular regions, with a sedentary breeding population of about 293–403 pairs [9], and is a regular migrant and partial winterer. In Basilicata, about 210–230 pairs are estimated [10], present in hilly areas (200–800 m above sea level) extending from the Agri and Basento Valleys to the south of the Apennines. The common buzzard (*Buteo buteo*) is a medium-sized diurnal bird of prey belonging to the Accipitridae family that has a high potential for adaptation to anthropological changes. In Italy it is a sedentary breeder, regular migrant, and winterer [11]. The common buzzard is widely distributed as a breeder throughout Italy, with widespread presence in mountainous and hilly regions from north to south, while it is more localized in the Po Valley, with gaps in its range at the Salento peninsula [12]. The breeding population is estimated between 2500 and 5000 pairs [13]. In Basilicata, the common buzzard occupies areas near watercourses in riparian wooded areas and open areas near the riverbed. The red kite and common buzzard are both predatory birds, and they can be called scavengers as they do not disdain carrion and habitually frequent landfills [14,15]. This feeding behavior puts the two species at risk for infection by endoparasites. The aim of this work was to report the discovery of *T. cati* larvae in the muscle tissue of two carcasses of birds of prey, a common buzzard (*Buteo buteo*) and a red kite (*Milvus milvus*), collected in the Basilicata Region, and to demonstrate that evidence of infection in these two birds makes them potential hosts capable of maintaining and spreading the parasite in the environment.

## 2. Materials and Methods

The red kite carcass was found in November 2020, while the common buzzard carcass was found in March 2021; both were collected in the province of Potenza, Basilicata Region, Southern Italy. The carcasses were sent to the Diagnostic Laboratory of the Istituto Zooprofilattico della Puglia e della Basilicata (IZSPB) for post-mortem investigations, and as part of the regional Wildlife Health Monitoring and Control Plan [16]. The two birds were subjected to chemical and toxicological analysis, as well as parasitological examination for *Trichinella* spp. Larvae. Pools of pectoral and tibial muscles were tested by enzymatic digestion, according to ISO 18743:2015 [17], using twenty grams for the red kite and thirteen grams for the common buzzard. In the red kite muscles, together with *Trichinella* larvae that were later identified as *T. pseudospiralis* [18], additional nematode larvae, differing from *Trichinella* in size and shape, were found. Identical nematode larvae were also present in the digestion fluid obtained from the buzzard tissue. The larvae collected from the two birds were observed under a light microscope and their size was measured. The larvae were then transferred in 90% alcohol and sent to the European Parasite Reference Laboratory (EURLP) of the Istituto Superiore di Sanità, (Rome, Italy) for species identification. DNA purification was carried out using the DNA IQ System and Tissue and Hair Extraction kit (Promega, Madison, WI, USA) according to manufacturer’s protocol. For molecular identification, specific PCR primers targeting the 18S rRNA gene [19] and the internal transcribed spacer I [20] were used, according to authors’ protocols. PCR products were purified using QIAquick PCR Purification Kit (Qiagen, Hilden, Germany) and sent to Eurofins Genomics (Ebersberg, Germany) for standard Sanger sequencing. The sequences were analyzed by CLC GenomicWorkbench (Qiagen, Hilden, Germany) and compared with the GenBank database for the species identification.

## 3. Results and Discussion

**Figure 1:**
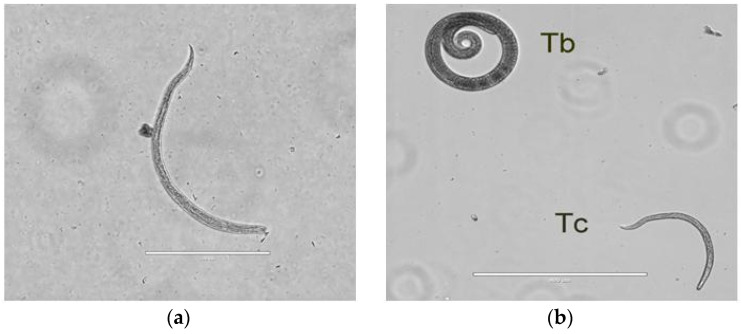
Larva of *Toxocara cati* isolated from common buzzard (*Buteo buteo*) (scale bar 200 µm) (**a**). Larva of *Toxocara cati* (TC) isolated from the red kite (*Milvus milvus*) compared with a larva of *Trichinella britovi* (TB) (scale bar 400 µm) (**b**).

Twenty-three (1.09 larvae per gram) and fifteen (1.15l pg) nematode larvae were recovered from the enzymatic digestion of pooled muscles of the red kite and common buzzard, respectively. The larvae collected from the two birds were identical, and measured about 400 µm in length and 15 µm in width, with a sub-terminal mouth in the anterior part of the body and a thinned tail at the distal end (Figure 1a). They were very different from *Trichinella* larvae, which had a larger size, lacked a mouth, and showed a larger and rounded posterior part. (Figure 1b). The molecular characterization performed at the EURLP identified the species as *T. cati* (Accession numbers: OM818648–OM818649–OM822766–OM822767).

In the last three years (2019–2021), many animal carcasses have been tested in our laboratory according to the Wildlife Health Monitoring and Control Plan, and the Trichinella Monitoring Plan, involving the territory of the Basilicata region. Out of 3439 animals, including 3401 wild boars (*Sus scrofa*), 13 wolves (*Canis lupus*), one wild cat (*Felix silvestris*), one marten (*Martes foina*), four otters (*Lutra lutra*), four badgers (*Meles meles*), two foxes (*Vulpes vulpes*), five common buzzards (*Buteo buteo*), two kites (*Milvus milvus*) and one griffon (*Gyps fulvus*), collected in different areas of the region, only the two raptors tested positive for *T. cati*. Data from the monitoring plans seem to indicate that the parasite is absent in the local wildlife, with the exception of the two birds. It is also possible that, due to their tiny size, *T. cati* will have escaped controls more focused on nematode larvae belonging to the *Trichinella* genus, or that the temperature (44–46 °C) and sedimentation time (30′) used for the detection of *Trichinella* may not have been optimal for recovery of *T. cati* larvae. Since both hosts are migratory birds, we cannot claim that the infection occurred in our territory, or in wintering areas. We also speculate that the two birds became infected either by ingesting contaminated embryonic eggs present in the soil or by ingesting other paratenic hosts, such as invertebrates and small rodents. 

To date, few authors have evaluated the natural infection and distribution of this parasite in birds; therefore, we believe that this topic needs further study. Although *T. cati* has already been detected in several wild birds [21,22], according to our knowledge, this is the first report of the occurrence of *T. cati* larvae in these two species, and there is no evidence of the presence of this parasite in the wildlife of the Basilicata region. 

The worldwide distribution of *T. cati* seems to be higher in domestic cat populations that have free access to the outdoor environment [23]. Furthermore, this parasite has been detected in several domestic and wild animals. A high prevalence has been reported in Eurasian lynxes (*Lynx lynx*) in Finland [24] and Poland [25], as well as in red lynxes (*Lynx rufus*) in the USA, [26]. Sporadic detections, such as larva migrans, have been reported in kiwi (*Apteryx mantelli*) [27] in New Zealand, in domestic land snail (*Rumina decollata*) specimens collected in the city of Buenos Aires [28], and in farmed chicken in Japan [29]. In Italy, *T. cati* larvae have been also detected in the muscle tissue of ostriches (*Struthio camelus*) and wild boar (*Sus scrofa*) [30], as well as in several wild birds such as the common kestrel (*Falco tinnunculus*), hen harrier (*Circus cyaneus*), and hooded crow (*Corvus cornix*) [21,22]. In most of these studies, *Toxocara* larvae were detected by enzymatic digestion of animal muscle tissue and subsequently identified at the species level by the amplification and sequencing of specific genes. Other studies were instead based on the morphological recognition of the parasite eggs, purified from soil or animal feces by the centrifugation–flotation technique [31].

## 4. Conclusions

In this study, we reported the first identification of *T. cati* larvae in the muscle tissue of a red kite and a common buzzard in Basilicata region. The identification of *T. cati* larvae in these two bird species hypothesizes the presence of new routes of transmission for this parasite, since birds are possible prey of wild animals, such as wolves (*Canis lupus*) and wild boars (*Sus scrofa*), which generally are not part of the life cycle of *T. cati*, and could provide for its survival in the environment. Deeper investigation is required to estimate the prevalence and distribution of *T. cati* in local wild fauna.

## Data Availability

Not applicable.
